# ATPase inhibitory factor 1 protects the heart from acute myocardial ischemia/reperfusion injury through activating AMPK signaling pathway

**DOI:** 10.7150/ijbs.64956

**Published:** 2022-01-01

**Authors:** Jia-Wei Wu, Hao Hu, Jin-sheng Hua, Li-Kun Ma

**Affiliations:** Department of Cardiology, The First Affiliated Hospital of USTC, Division of Life Science and Medicine, University of Science and Technology of China, Hefei, 230001, China

**Keywords:** ATPase inhibitory factor 1, ischemia/reperfusion injury, heart, AMPK

## Abstract

**Rationale:** Myocardial ischemia/reperfusion (I/R) injury is a common clinic scenario that occurs in the context of reperfusion therapy for acute myocardial infarction (AMI). The mitochondrial F1Fo-ATPase inhibitory factor 1 (IF1) blocks the reversal of the F1Fo-ATP synthase to prevent detrimental consumption of cellular ATP and associated demise. In the present study, we study the role and mechanism of IF1 in myocardial I/R injury.

**Methods:** Mice were ligated the left anterior descending coronary artery to build the I/R model *in vivo*. Rat hearts were isolated and perfused with constant pressure according to Langendorff. Also, neonatal cardiomyocytes hypoxia-reoxygenation (H/R) model was also used. Myocardial infarction area, cardiac function, cellular function, and cell viability was conducted and compared.

**Results:** Our data revealed that IF1 is upregulated in hearts after I/R and cardiomyocytes with hypoxia/re-oxygenation (H/R). IF1 delivered with adenovirus and adeno-associated virus serotype 9 (AAV9) ameliorated cardiac dysfunction and pathological development induced by I/R *ex vivo* and *in vivo*. Mechanistically, IF1 stimulates glucose uptake and glycolysis activity and stimulates AMPK activation during *in vivo* basal and I/R and *in vitro* OGD/R conditions, and activation of AMPK by IF1 is responsible for its cardioprotective effects against H/R-induced injury.

**Conclusions:** These results suggest that increased IF1 in the I/R heart confer cardioprotective effects via activating AMPK signaling. Therefore, IF1 can be used as a potential therapeutic target for the treatment of pathological ischemic injury and heart failure.

## Introduction

Acute myocardial infarction (AMI) caused by coronary occlusion is the main cause of disability and death worldwide. After AMI, a large number of dead cardiomyocytes are replaced by fibrous scar tissue, leading to ventricular remodeling and heart failure (HF) 1, 2. In order to rescue ischemic myocardium and limit infarct size, timely and effective reperfusion is the current treatment option for patients with AMI. However, this leads to additional injury, known as ischemia/reperfusion (I/R) injury 3, 4. Therefore, it is necessary to identify the methods to protect cardiomyocytes from I/R injury in the early stage of reperfusion and to elucidate the potential protective mechanisms to promote the development of new therapeutic methods for ischemic heart disease.

The efficiency of the mitochondrial F1Fo-ATP synthase that generates large amounts of ATP guaranteed energy homeostasis of mammalian cells. ATPase inhibitory factor 1 (IF1) is a protein that binds with F1F0-ATP synthase 5-7. It was reported that when F1F0-ATP synthase reverses its function and hydrolyzes ATP, IF1 binds and inhibits ATP synthase with a large drop in the inner membrane potential 8, 9. Thus, IF1 prevents total ATP depletion and consequent cell death during hypoxia or starvation, a function which is essential under pathologic conditions, such as cardiac ischemia 10, 11. This is consistent with the report that endogenous IF1 overexpression is involved in the reprograming of energy metabolism to enhance glycolysis by limiting ATP production by F1F0-ATP synthase in both cell culture and tissue-specific mouse models 12, 13. This promotes the production of mitochondrial reactive oxygen species (ROSs) that modulate various signaling pathways favoring cellular processes 14. Many studies have evaluated the physiologic role and gene expression of endogenous IF1, in spite of this, the underlying molecular events by which IF1 could be protective in I/R injury has not been yet properly addressed.

AMPK is a stress response kinase, which can regulate a variety of important physiological and metabolic processes, including energy homeostasis, cell apoptosis, and cell metabolism 15-18. It has been demonstrated that AMPK can up-regulate cellular antioxidant enzyme systems, thereby reducing oxidant-induced damage in I/R injury 19. Therefore, this study aims to investigate the cardioprotective effect of IF1 on I/R injury and whether AMPK activation is involved in the protective effect of IF1.

## Methods and materials

**Animals.** Adult male C57BL/6 mice from (Shanghai Slac Laboratory Animal Co. Ltd.) were nursed according to the Guidelines for the Care and Use of Laboratory Animals published by the US National Institutes of Health (NIH Publication, 8th Edition, 2011), and the animal procedure was approved by the Institutional Review Board of The First Affiliated Hospital of USTC.

**The mouse I/R injury model.** Mice were fasting overnight before surgery. 50 mg/kg pentobarbital sodium was injected intraperitoneally. Injected and fixed in the lateral position on the operating table. The fourth intercostal space was opened to expose the heart, and the left anterior descending artery (LAD) was sutured with silk thread on the inflated balloon. After 30 minutes, LAD was reperfused by deflation and balloon withdrawal. Echocardiography was carried out in each animal after the conduction of myocardial IR with an ultrasound machine (Vevo 2100, USA).

### Adenovirus and adeno-associated virus vector construction, infection, and injection

Recombinant adenoviruses expressing mouse IF1 (Ad-IF1) and NC (Ad-NC) were prepared as previously described 20. The AAV9 expressing IF1 (AAV9-IF1) and control (AAV9-Ctrl) were constructed as described previously 21. Surgery and adenovirus delivery was performed as previously described. In short, mice anesthetized with pentobarbital sodium (50 mg/kg, i.p.) underwent thoracotomy. A 27-G needle containing 100 μL of diluted adenovirus (3 x 10^10^ pfu/mL) or sterile saline was injected into left ventricle. When the solution was injected, the aorta and pulmonary aorta were clamped at the distal end of the syringe for 10 s, and then the chest was closed. The hemodynamics of the heart was studied on the 4th day after adenovirus injection. AAV9 was injected into adult mice by using a 29-gauge insulin syringe (BD, Ultral fine needle) as described previously 21. 6-week-old C57BL/6 mice were injected with 100 μl containing 1x10^12^ vg AAV9-NC or 200 μl containing 3 × 10^12^ vg AAV9-IF1 viruses using a 29-gauge insulin syringe (BD, Ultral fine needle) through tail vein. 3 weeks after AAV injection, hearts were treated with I/R injury *in vivo* (30min/24h). The heart function, infarct size, LDH level, and serum cTnT level were measured after 24 hours.

### Isolation, culture, and adenoviral infection of neonatal rat cardiomyocytes (NRCMs)

NRCMs were isolated from neonatal S-D rat hearts. After 2 h of culture to achieve myocytes attachment, adenovirus was added at a multiplicity of infection (MOI) of 100 for 2 h. All experiments were performed after 36 h of infection.

### I/R injury model in Langendorff-perfused mouse hearts

After anesthetizing the mice with pentobarbital sodium (50 mg/kg i.p.), the hearts were removed quickly and perfused with Krebs-Henseleit solution at 37℃ using a Langendorff apparatus as previously described 20. The development pressure of LV (LVDP), LVEDP and the maximum rate of pressure development or decay with time (+dP/dtmax) were evaluated with the PowerLab system (AD Instrument) by powerlab system (AD instrument). At the end of reperfusion, the heart was quickly removed and frozen in liquid nitrogen for further analysis.

### TUNEL assay

Fixed cultured cells were incubated with a TUNEL stain (*In situ* Cell Death Detection Kit, POD, Roche Diagnostics GmbH, Mannheim, Germany) and co-stained with Hoechst 33342 and visualized using a Leica SP5 confocal microscopy system. The images were analyzed using ImageJ software.

### Infarct size measurement

The isolated mice hearts subjected to 30 min of ischemia followed by 1h of reperfusion *ex vivo* and mice hearts subjected to 30 min of ischemia followed by 24 h of reperfusion *in vivo* were frozen, and the LV tissue was cut into 5 slices. The slices were incubated in 1% TTC for 20 min Infarct size was calculated using Image-J software, and the infarct area was expressed as a percentage of area at risk (ARR). Cardiac function was evaluated by echocardiography using VEVO 2100 (Visual Sonics) under anesthesia with 1.5% isoflurane. For fibrosis measurement, Masson's Trichrome staining was performed.

### Immunoblotting analysis

The LV tissue and the cardiomyocytes were lysed by RIPA buffer with protein inhibitors mixture as described previously 22. The protein fractions were analyzed by standard immunoblotting when transferred to polyvinylidene fluoride membrane (BioRad) and probed with relevant antibodies. Relative level of proteins was determined by antibodies against IF1 (1:2000), b-actin (1:4000), phospho-AMPK (Thr172, 1:1000), AMPK (1:2000), and Cleaved-caspase 3 (1:1000). Antibodies were purchased from Cell Signaling Technology.

### Statistical analysis

Data were expressed as means ± SEM and performed with Graphpad Prism software (version 8.0). For multiple comparisons, ANOVA or repeated ANOVA followed by the LSD *post-hoc* test was used. A P-value of p < 0.05 was considered statistically significant.

## Results

### IF1 is downregulated during I/R and H/R

**Figure 1:**
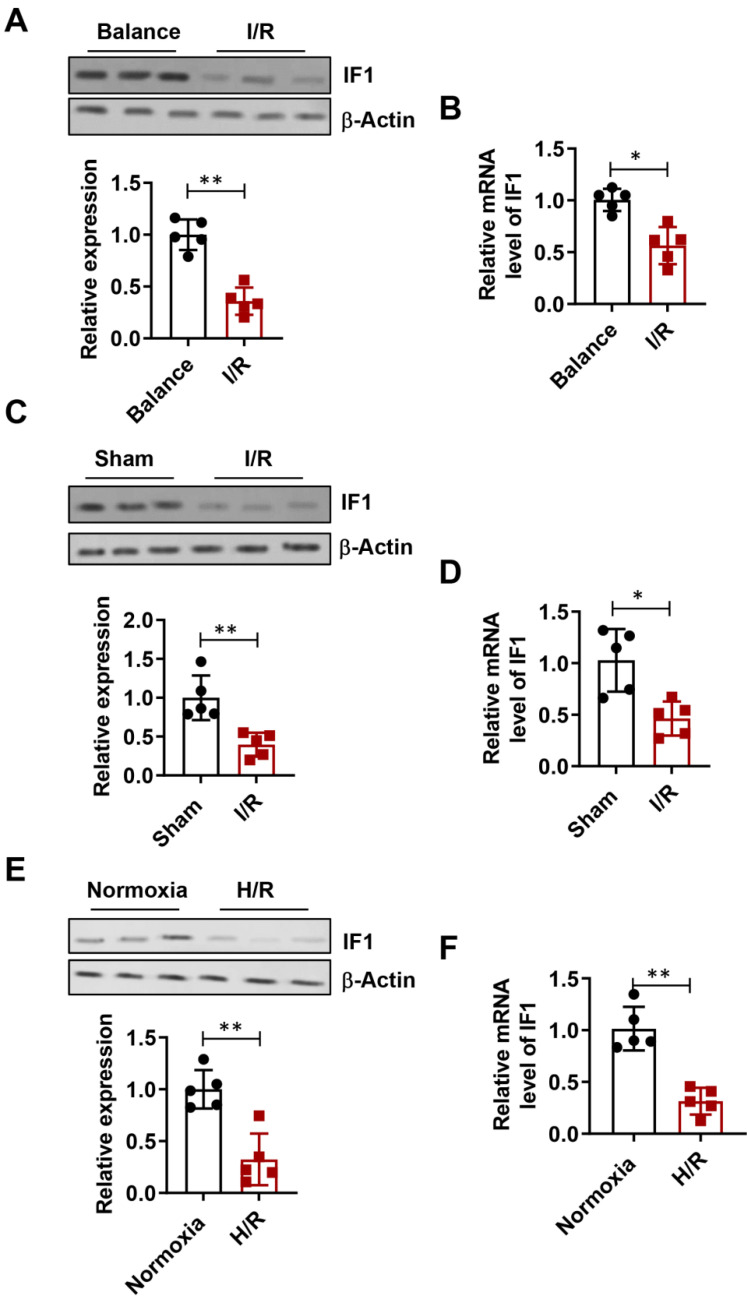
IF1 protein expression is downregulated during I/R and H/R. A, Immunoblotting analysis of IF1 protein in isolated perfused I/R (30/45 min) hearts. β-Actin was used as an internal reference. B, Q-PCR analysis of IF1 mRNA in isolated perfused I/R (30/45 min) hearts; C, Immunoblotting analysis of IF1 protein in I/R (30min/24h) hearts; D, Q-PCR analysis of IF1 mRNA in I/R (30min/24h) hearts; E, Immunoblotting analysis of IF1 protein in simulated I/R (20/30 min) cardiomyocytes. F, Q-PCR analysis of IF1 mRNA in simulated I/R (20/30 min) cardiomyocytes. n = 5 each. *P <0.05, **P<0.01.

The expression level of IF1 in isolated perfused I/R (30 min/1 h) hearts (Fig. 1A), I/R (30min/24h) hearts (Fig. 1C), simulated I/R (30 min/1 h) cardiomyocytes (Fig. 1E) were analyzed. IF1 protein levels were downregulated by I/R injury in hearts. Similar expression pattern of IF1 mRNA was observed in the I/R cardiomyocytes and hearts (Fig. 1B, D and F). These results suggest a possible involvement of IF1 in I/R injury.

### Effect of IF1 overexpression with adenovirus encoding IF1 on cardiac function and LDH release during I/R *ex vivo*

**Figure 2:**
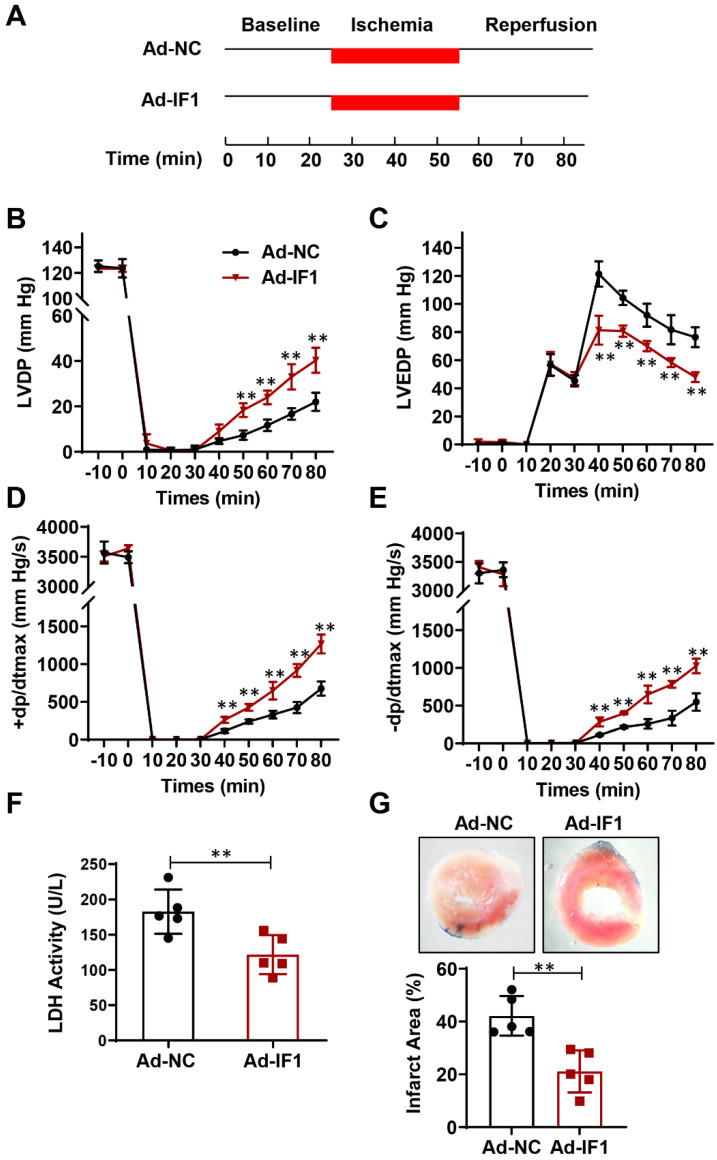
Effect of IF1 overexpression with adenovirus encoding IF1 on cardiac function and LDH release during I/R *ex vivo*. A, Schematic of I/R stimulation and IF1 overexpression with adenovirus. B, Time course of LVDP (B), LVEDP (C), and +dP/dt_max_ (D) and 2dP/dt_max_ (E); n = 5 each. F, Fold changes of lactate dehydrogenase (LDH) activities in Ad-IF1 group compared with the Ad-NC group. n = 4-5. G, Representative images and analysis of the infarct size in isolated I/R (30 min/2 h) hearts; n = 5 each. **P<0.01.

We then studied the role of IF1 in cardiac I/R injury with adenovirus-mediated IF1 overexpression (Ad-IF1) (Fig. 2A). Western blot analysis showed that IF1 was significantly overexpressed in the hearts of mice infected with Ad-IF1 (Fig. S1A). There are no significant differences were observed in LVDP (Fig. 2B), LVEDP (Fig. 2C), ± dP/dt (Fig. 2D and E) between the Ad-NC control and Ad-IF1 groups during the pre-ischaemic phase in Langendorff perfused mice hearts, while I/R-induced contractile dysfunction (Fig. 2B-E) in Ad-NC hearts were significantly improved by Ad-IF1. Meanwhile, I/R-induced increases of LDH activity in coronary perfusate after 45 min of reperfusion as well as infarct size after 2 h of reperfusion (Fig. 2F and G) were significantly decreased by Ad-IF1. These results suggest that a higher level of IF1 is needed for cardiac repair after I/R injury, and the change of IF1 in a certain level does not affect basic myocardial function.

### Effect of IF1 overexpression with AAV9 vector encoding IF1 on infarct size and cardiac function during myocardial I/R (mice)

**Figure 3:**
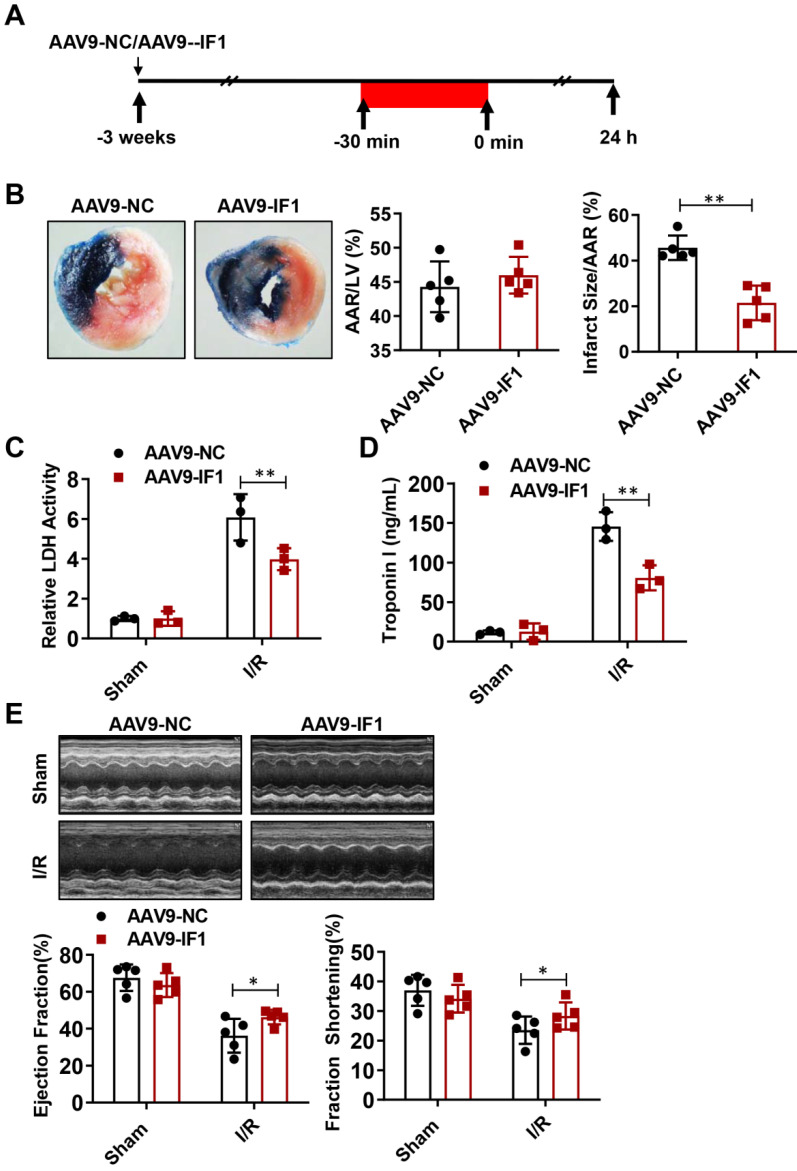
Effect of IF1 overexpression with AAV9 vector encoding IF1 on infarct size and cardiac function during myocardial I/R (mice). A, Schematic of *in vivo* I/R stimulation and IF1 overexpression with AAV9. B, Representative and quantification of 2, 3, 5-triphenyte-trazoliumchloride (TTC)/Evans blue staining for infarct size in the hearts at 24 hours post-I/R. n = 5. C, Fold changes of LDH activities in AAV9-IF1 group compared with the AAV9-NC group. n = 3. D, Serum cTnI ELISA analysis in AAV9-IF1 group compared with the AAV9-NC group. n = 3. E, left ventricle (LV) ejection fraction (LVEF), and LV fractional shortening (LVFS) measured by echocardiography (Echo). n = 5. *P < 0.05, **P < 0.01.

To determine whether IF1 contributes to the recovery of myocardial I/R injury, we overexpressed IF1 in the heart by tail vein injection of AAV9-IF1 at 3 weeks before the myocardial I/R (Fig. 3A). Western blot analysis showed that IF1 was significantly overexpressed in the hearts of mice infected with AAV-IF1 (Fig. S1B). We first analyzed whether AAV9-IF1 decrease infarct size at day 1 post-I/R. The I/R-insulted hearts with AAV9-NC and AAV9-IF1 infected have similar AAR to LV (Figure 3B), while the infarct area (Fig. 3B), serum LDH activity (Fig. 3C), and serum Troponin I level (Fig. 3D) were all significantly reduced in the AAV9-IF1 group compared with these in the AAV9-NC group. We next analyzed the long-term protective effects of AAV9-IF1 in the I/R hearts. The mice injected with AAV9-NC and AAV9-IF1 showed comparable LVEF and LVFS with these in the Sham groups, whereas the AAV9-IF1 treatment significantly improved these parameters worsened in the I/R control group at the 4-week after I/R (Fig. 3E). Notably, the fibrotic area was significantly smaller in AAV9-IF1-infected hearts than in controls at 4 weeks post I/R (Figure S3A). Therefore, IF1 may function as an endogenous protective factor in the heart against I/R injury.

### IF1 is involved in the response to H/R-induced injury in cardiomyocytes

**Figure 4:**
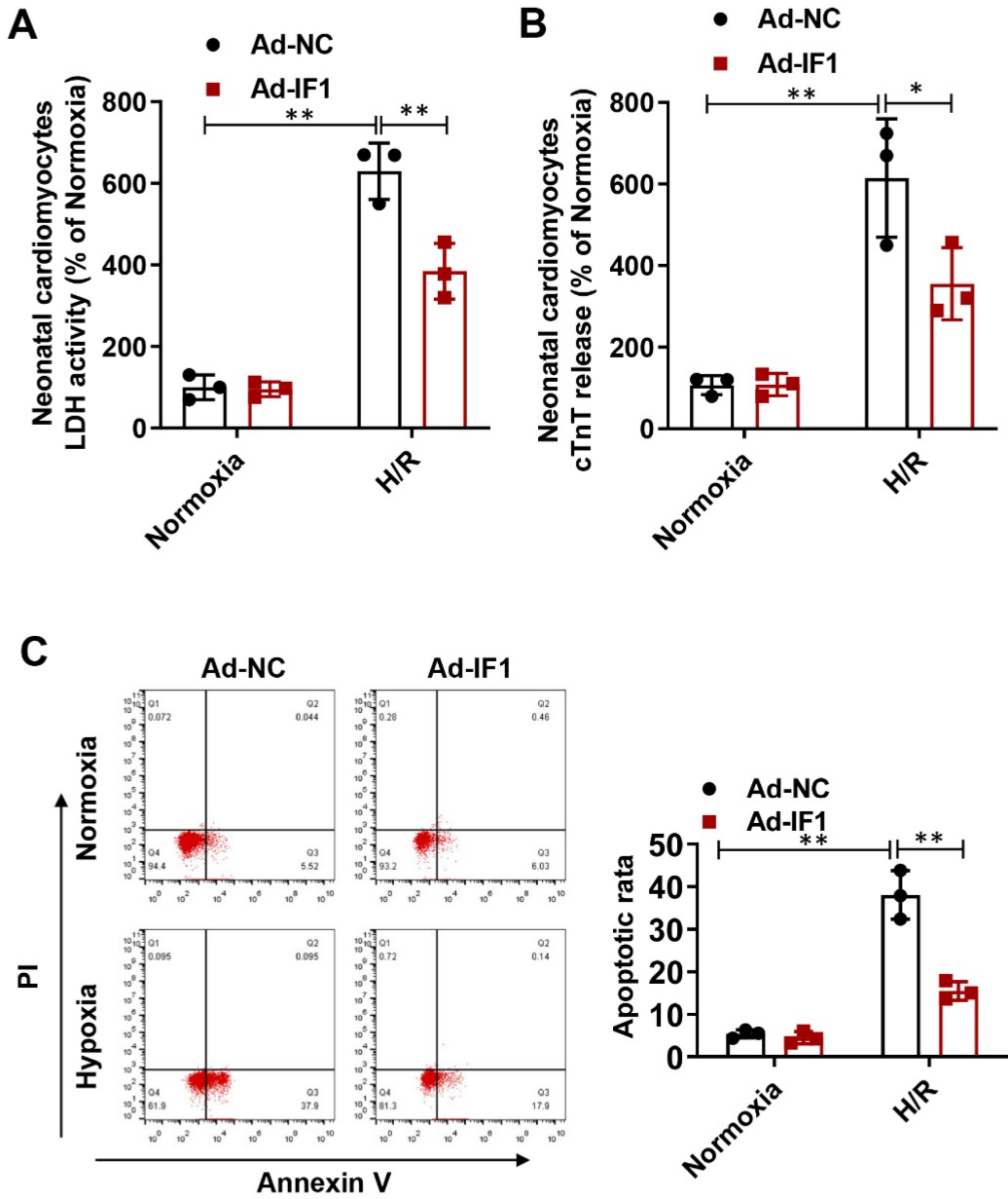
IF1 is involved in the response to H/R-induced injury (cTnT and LDH release, apoptosis) in cardiomyocytes. A, LDH released from NRCMs at the 1 hour of reperfusion. B, cTnT released from NRCMs induced by H/R injury. C, Representative flow cytometric assessment of apoptosis via Annexin V/PI staining and Flow cytometric quantification. n = 3. *P <0.05, **P<0.01.

To further explore whether the protective effects of IF1 against I/R injury is related to cardiomyocytes, we performed H/R injury model on NRCMs. We found the H/R injury-increased LDH release (Fig. 4A) at the 1 hour of reperfusion was decreased by Ad-IF1. Concomitantly, Ad-IF1 significantly decreased the cTnT release induced by H/R injury (Fig. 4B). Similar results were found by Annexin V/PI staining, that is, the proportion of the right quadrant cells was increased in the H/R-treated NRCMs and recovered with Ad-IF1 (Fig. 4C). Moreover, IF1 knockdown aggravated H/R-induced cardiomyocytes injury, as evidenced by increased LDH release, cTnT release, and increased number of apoptotic cells (Fig. S2). Therefore, IF1 is an endogenous protective factor for cardiomyocytes against I/R injury.

### IF1 stimulates glucose uptake and glycolysis activity

**Figure 5:**
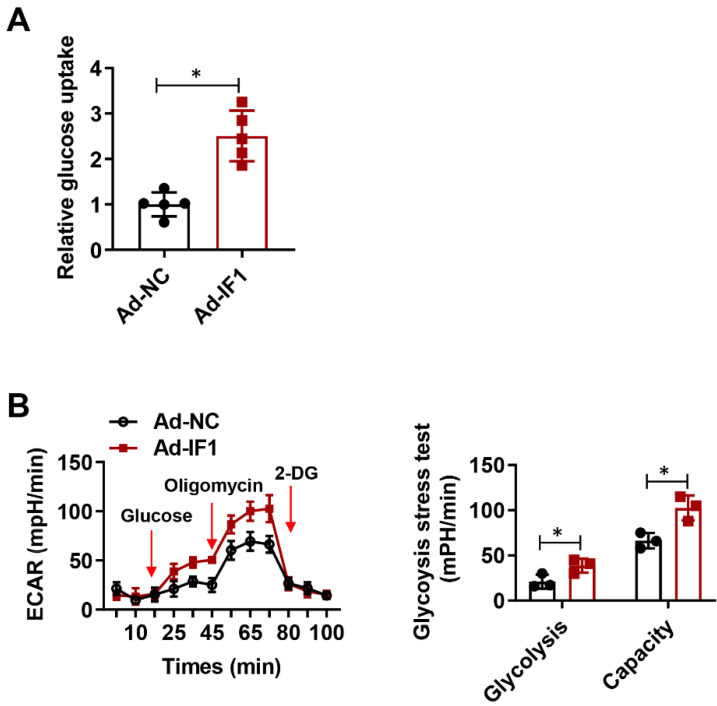
IF1 stimulates glucose uptake and glycolysis activity. A, Glucose uptake assay. n = 3. B, Glycolytic and mitochondrial respiration measurement with Seahorse Extracellular Flux Analyzer, with ECAR to reflect aerobic glycolysis. n = 5. *P <0.05.

IF1 is reported to exert its cardioprotective effects in the I/R injury whereas ATP wastage accounts for a large proportion of the bio-energetic damage, but whether by overexpression IF1 to target the F1Fo-ATPase is protective or not in I/R injury has not been yet properly addressed. To determine whether IF1 could save energy consumption after I/R injury, we performed glucose uptake assay and glycolytic and mitochondrial respiration measurement with Seahorse Extracellular Flux Analyzer, with ECAR to reflect aerobic glycolysis. IF1 overexpression remarkably promotes glucose uptake (Fig. 5A), increased glycolysis and glycolytic capacity, which was reflected by ECAR measurement (Fig. 5B).

### IF1 stimulated AMPK activation during *in vivo* basal and I/R and *in vitro* OGD/R conditions, and activation of AMPK by IF1 is involved in its cardioprotection against H/R-induced injury

**Figure 6:**
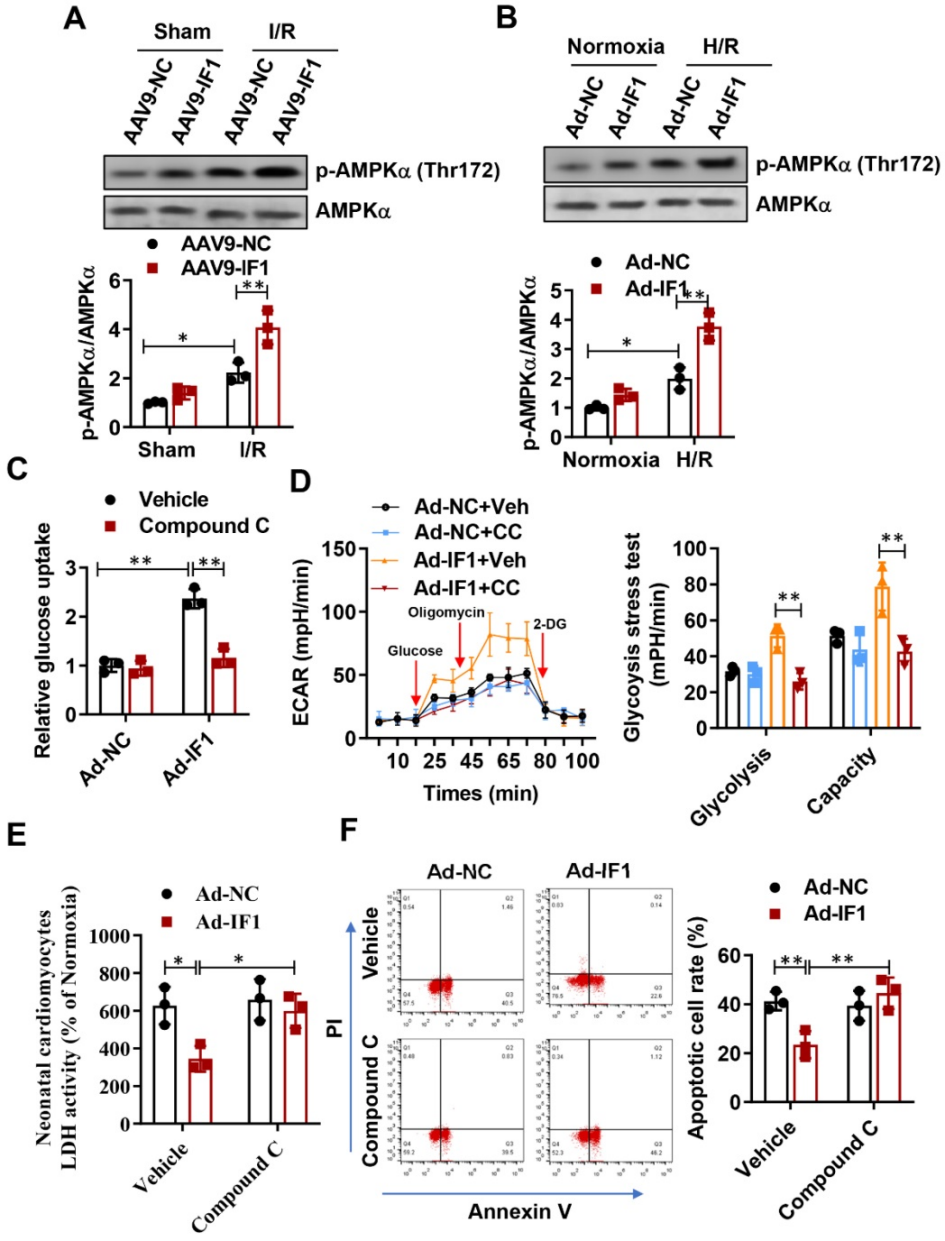
IF1 confer cardioprotection via stimulating AMPK activation. A, Western blot analysis of phosphorylation levels of AMPK at Thr-172 in the heart. B, Western blot analysis of phosphorylation levels of AMPK at Thr-172 in cardiomyocytes. C, Glucose uptake assay and glycolytic and mitochondrial respiration (D) measurement with Seahorse Extracellular Flux Analyzer. E, LDH released from NRCMs at the 1 hour of reperfusion. F, Representative flow cytometric assessment of apoptosis via Annexin V/PI staining and Flow cytometric quantification. n = 3 each. *P <0.05, **P<0.01.

AMPK activation in the heart is protective against cardiac ischemic injury 23-25. Thus, the activating phosphorylation levels of AMPK at Thr-172 in the heart were assessed. Phosphorylation levels of AMPK at Thr-172 in the heart were significantly increased after I/R, while systemic delivery of IF1 by AAV9 resulted in a more increase in AMPK phosphorylation in the ischemic heart following I/R injury (Fig. 6A). Furthermore, we also observed this phenomenon on H/R-induced injured NRCMs, where H/R-induced injury increased AMPK phosphorylation in the cardiomyocytes and treatment of cardiac myocytes with IF1 by adenovirus enhanced the phosphorylation of AMPK (Fig. 6B). Next, we examined whether AMPK phosphorylation mediate the regulation of IF1 on glucose uptake and glycolysis activity. In cardiomyocytes, pretreatment of compound C markedly decreased IF1-induced AMPK phosphorylation (Fig. S1C). Our data indicated that Ad-IF1 increased glycolysis and glycolytic capacity, while pretreatment of AMPK inhibitor Compound C abolished these effects (Fig. 6C and D). To further determine whether the cardioprotective effects of IF1 on cardiomyocyte is mediated by AMPK, we induced H/R injury in the NRCMs and infected with Ad-IF1 with or without treatment of Compound C and measured LDH release and cell apoptosis. Ad-IF1 decreased H/R injury-increased LDH release and cardiomyocytes apoptosis at the 1 hour of reperfusion while Compound C abolished these cardioprotective effects (Fig. 6E, F and Fig. S4).

### Compound C abrogated IF1-afforded cardioprotection *in vivo*

**Figure 7:**
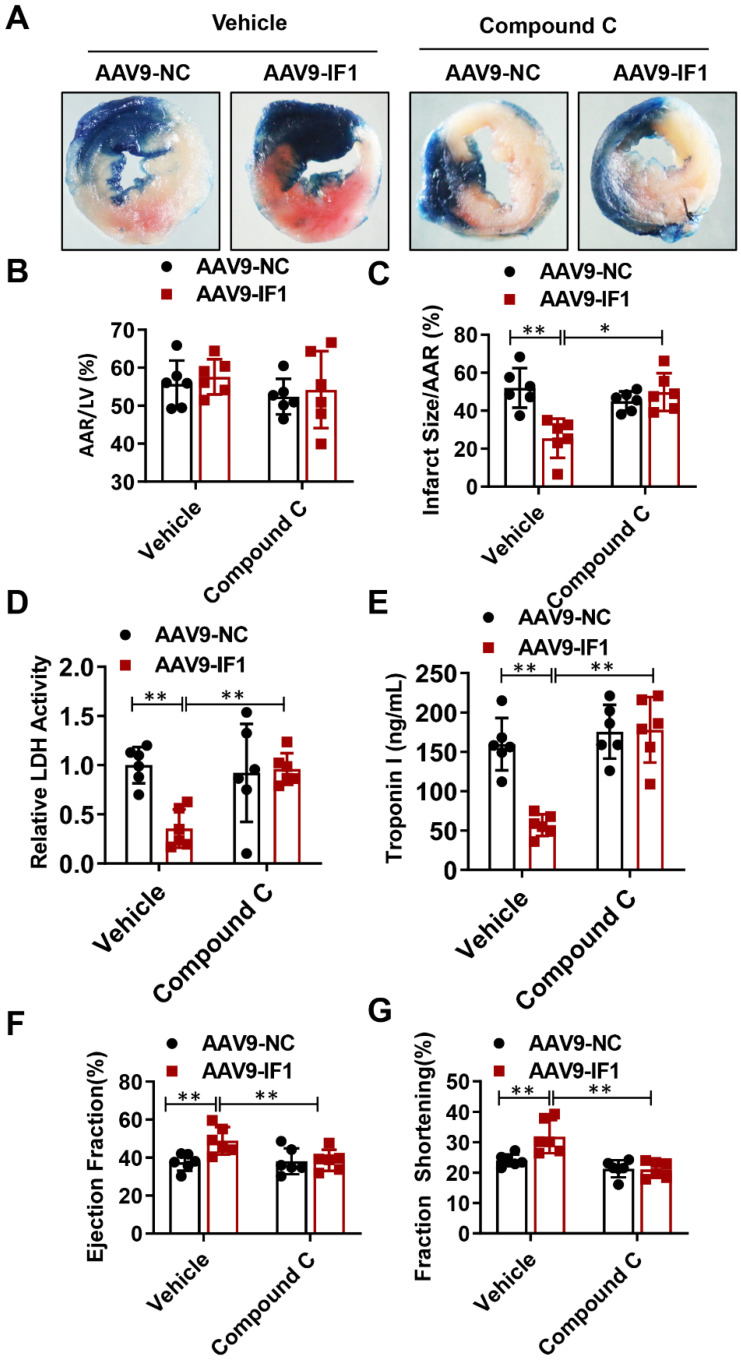
Compound C abrogated IF1-afforded cardioprotection. A-C, Representative images, and analysis of the infarct size in isolated I/R (30 min/2 h) hearts. D, serum LDH activity analysis. E, Serum Troponin I level analysis. F-G, left ventricle (LV) ejection fraction (LVEF) and LV fractional shortening (LVFS) measured by echocardiography (Echo). n = 6. *P <0.05, **P<0.01.

To further determine whether IF1 contributes to the alleviation of myocardial I/R injury through AMPK signaling pathway, we overexpressed IF1 in the heart and treat the I/R mice at the beginning of reperfusion with Compound C. Although AAV9-NC and AAV9-IF1 groups showed comparable AAR to LV after I/R (Fig. 7A, B), the infarct area (Fig. 7C), serum LDH activity (Fig. 7D) and serum Troponin I level (Fig. 7E) were significantly attenuated in the AAV9-IF1 group compared with these in the AAV9-NC group without Compound C, while these effects were all abrogated by Compound C treatment. At 4 weeks post-I/R, treatment with Compound C almost completely abolished IF1-improved cardiac function, as evidenced by reduced LVEF and LVFS (Fig. 7D-7E). Meanwhile, cardiac fibrosis was mitigated by AAV-IF1, which was reversed with Compound C treatment, as evaluated by Masson's Trichrome staining (Fig. S3B). Therefore, AMPK mediates the cardioprotective effects of IF1 against I/R injury *in vivo*.

### IF1 activates ROS as an AMPK upstream pathway

**Figure 8:**
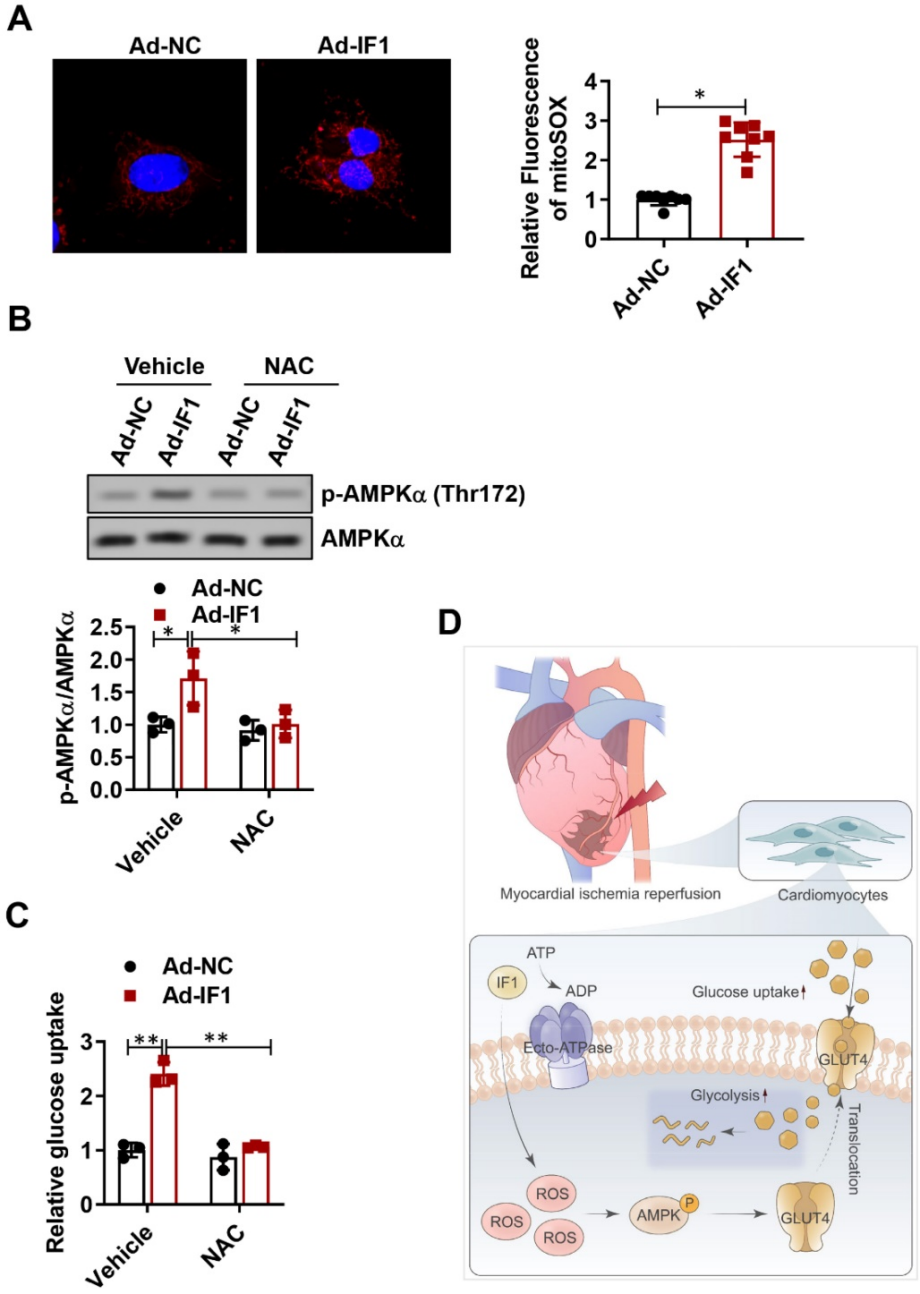
IF1 activates ROS as an AMPK upstream pathway. A, following infection of NRCMs for 48h with Ad-IF1, ROS were stained with an ROS-sensitive dye DCF-DA. B, Western blot analysis of phosphorylation levels of AMPK. C, NAC blocked IF1-mediated glucose uptake. n = 3. D, The graphic abstract of the study. *P <0.05, **P<0.01.

ROS has been shown to be associated with AMPK activation (30, 31). Thus, we attempted to determine whether the release of ROS is involved in IF1-induced AMPK phosphorylation. Following infection of NRCMs for 48h with Ad-IF1, there was an increased intensity of staining with DCF-DA, a ROS-sensitive dye (Fig. 8A). Moreover, ROS chelation using NAC, a ROS scavenger, resulted in decreased AMPK phosphorylation (Fig. 8B). In addition, NAC blocked IF1-mediated glucose uptake (Fig. 8C), demonstrating that IF1 increases AMPK phosphorylation through ROS generation.

## Discussion

In the present study, we study the role and mechanism of IF1 in myocardial I/R injury. First, our data indicated that IF1 protein expression is downregulated during I/R and H/R. Second, IF1 delivered with adenovirus and AAV9 protected against cardiac dysfunction and pathological development induced by I/R *ex vivo* and *in vivo*. Third, IF1 stimulates glucose uptake and glycolysis activity and stimulates AMPK activation during *in vivo* basal and I/R and *in vitro* OGD/R conditions. Finally, we found activation of AMPK by IF1 is involved in its cardioprotection against H/R-induced injury. These results indicate that upregulation of IF1 in the I/R heart exerts its cardioprotection by activating AMPK signaling pathway.

It has been reported that the main function of IF1 is to reduce the decrease of ATP by inhibiting the hydrolysis activity of ATP synthase under myocardial ischemia 7-9, 26. Inhibiting ATP hydrolysis has been proposed as a potential way for the treatment of various pathological conditions, including cardiac I/R injury 10, 27. In preclinical models, IF1 mimetic compounds have been shown to enhance the cardiac performance of isolated hearts subjected to myocardial ischemia/reperfusion 28-30. Inhibition of ATP hydrolysis has been proposed as a potential clinical strategy for the treatment of various pathological conditions, including myocardial ischemia/reperfusion injury [10, 26]. IF1 mimics, such as bms-199264 and bms-250685, have been shown to enhance the cardiac performance of isolated hearts subjected to myocardial ischemia/reperfusion [27-29]. Before identifying IF1 as a therapeutic target for heart disease, it is necessary to obtain the functional role of IF1 in pathological heart. In this study, we further explored the protective effect of IF1 delivered by adenovirus and AAV9 and found that IF1 overexpression protects the heart from I/R-induced cardiac dysfunction and pathological development *in vitro* and *in vivo*.

There are many literatures that activation of pro-survival kinases, such as ERK and AMPK, can achieve strong cardiac protection in myocardial reperfusion 4, 20, 31. Activation of myocardial AMPK has been shown to display salutary effects on ischemic heart disease and glucose homeostasis 32-36, we, therefore, hypothesized that the AMPK signaling pathway is primarily involved in the protective effects of IF1 as an underlying mechanism. At the cellular level, AMPK activation was found to result from ROS production induced by IF1 treatment. ROS-induced AMPK activation might be involved in the observed hypoglycemic effects of IF1. It is widely accepted that AMPK phosphorylation increases glucose uptake through the enhanced GLUT4 translocation from the cytoplasmic fraction to the plasma membrane 37-40; therefore, AMPK activation induced by IF1 could increase glucose uptake in these mice, ultimately eliciting glucose-lowering effects. This was confirmed by our *in vitro* results that IF1 significantly increased glucose uptake in NRCMs. Glucose uptake was attenuated after the inactivation of the AMPK pathway using AMPK-specific silencing by the AMPK inhibitor compound C. Thus, the IF1/AMPK signaling axis may contribute to the cardioprotective effects. Collectively, the therapeutic approaches to enhance IF1 production can be valuable for the prevention or treatment of I/R injury. A previous study suggests that in mammalian neurons mitochondria adapt to respiratory stress by upregulating IF1, which exerts a protective role by coordinating pro-survival cell mitophagy and bioenergetics resilience^41^. Therefore, it is of great interest to investigate whether IF1 confer cardioprotective effects via activating mitophagy.

In conclusion, in the present study, we showed that IF1 is downregulated in mouse hearts under I/R stress, IF1 delivered by adenovirus and AAV9 protected the hearts from I/R injury. Mechanistically, we demonstrated that activation of AMPK by IF1 is involved in the cardioprotection of IF1 against I/R injury (Fig. 8D). These results suggest that IF1 delivery may serve as a potential therapeutic strategy in treating pathological cardiac ischemic injury and heart failure.

## Supplementary Material

Supplementary figures.
