# Supplementary material for: ATPase inhibitory factor 1 protects the heart from acute myocardial ischemia/reperfusion injury through activating AMPK signaling pathway
Source: Int J Biol Sci. 2022 Jan 1;18(2):731–41. doi: 10.7150/ijbs.64956 (PMC8741848; doi:10.7150/ijbs.64956)
Supplement: Supplementary file 1 — Supplementary figures. [file ijbsv18p0731s1.pdf]

Supplemental Figure 1

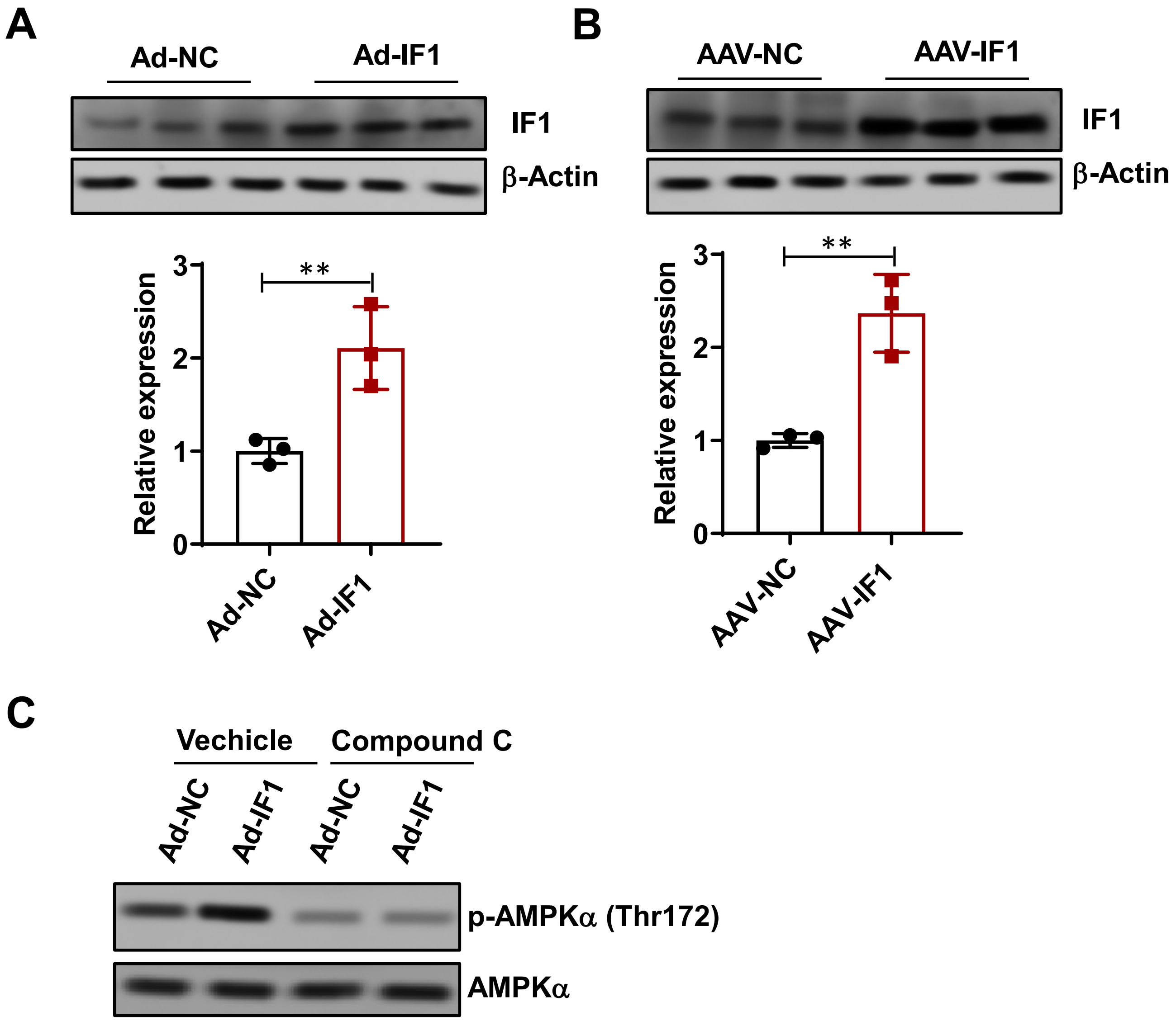

Supplemental Fig.1 Western blot analysis for overexpression efficiency of Ad-IF1 (A) and AAV-IF1 in hearts (B), as well the inhibitory effect of compound C on AMPK (C). n = 3 each. **P<0.01.

Supplemental Figure 2

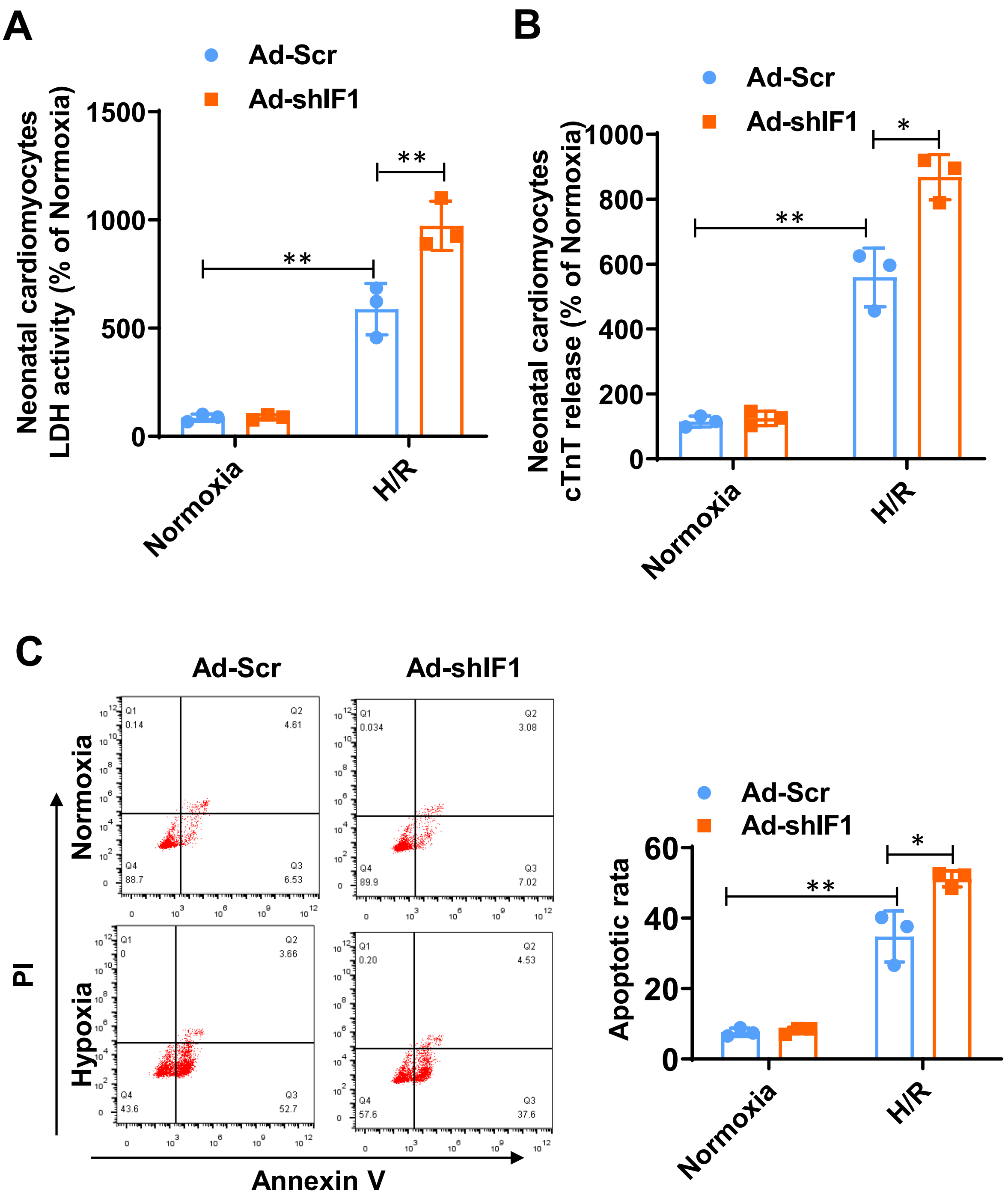

Supplemental Fig.2 IF1 knockdown aggravated H/R-induced cardiomyocytes injury. A, LDH released from NRCMs at the 1 hour of reperfusion. B, cTnT released from NRCMs induced by H/R injury. C, Representative flow cytometric assessment of apoptosis via Annexin V/PI staining. n = 3 each. *P <0.05, **P<0.01.

Supplemental Figure 3

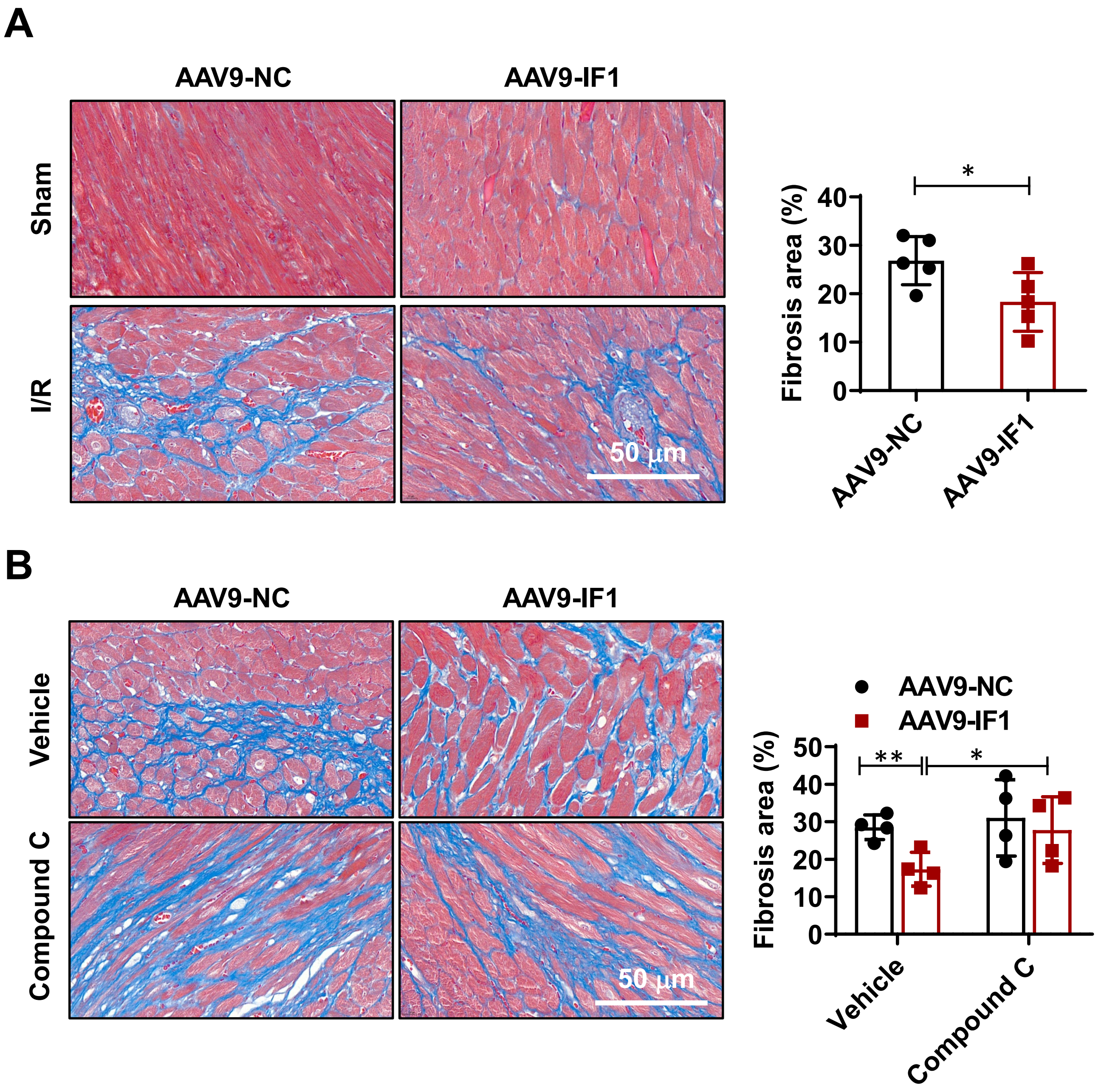

Supplemental Fig.3 Cardiac fibrosis was evaluated via Masson's Trichrome staining. A. Representative images and quantitative data of hearts stained with Masson's trichrome in AAV9-IF1 group compared with the AAV9-NC group. n = 5 each. B. Representative images and quantitative data of hearts infected with Masson's trichrome. Scale bar = 50 μm. n = 4 each. **P*<0.05, ***P*<0.01.

Supplemental Figure 4

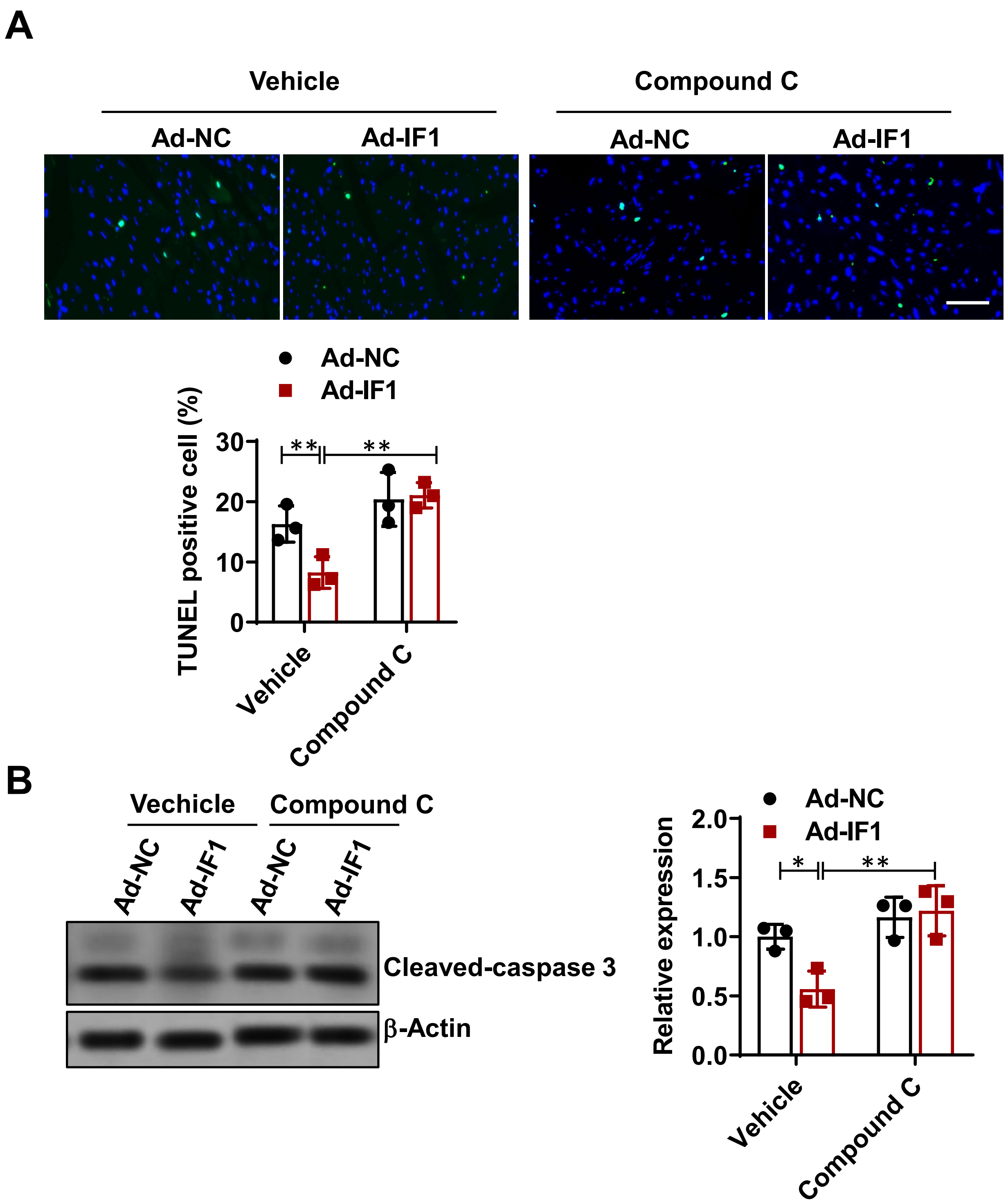

Supplemental Fig.4 The effect of IF1 and compound C on cardiomyocyte apoptosis. A. Immunostaining of TUNEL in cardiomyocytes. The results are displayed as representative images (upper, scale bar = 20 μm) and as a bar chart of TUNEL-positive cells. B. Representative immunoblots of cleaved-caspase 3 and quantitative analysis. n = 3 each. **P*<0.05, ***P*<0.01.
